# Altmetric coverage of health research in Ireland 2017-2023: a protocol for a cross-sectional analysis

**DOI:** 10.12688/hrbopenres.13895.3

**Published:** 2024-10-22

**Authors:** Melissa K Sharp, Patricia Logullo, Pádraig Murphy, Prativa Baral, Sara Burke, David Robert Grimes, Máirín Ryan, Barbara Clyne

**Affiliations:** 1 Department of Public Health and Epidemiology, RCSI University of Medicine and Health Sciences, Dublin 2, Ireland; 2 Centre for Statistics in Medicine, EQUATOR Network UK Centre, University of Oxford, Oxford, England, UK; 3 School of Communications, Dublin City University, Dublin, Leinster, Ireland; 4 Department of International Health, Johns Hopkins Bloomberg School of Public Health, Baltimore, Maryland, USA; 5 Centre for Health Policy and Management Discipline of Public Health and Primary Care, The University of Dublin Trinity College, Dublin, Leinster, Ireland; 6 School of Medicine, The University of Dublin Trinity College, Dublin, Leinster, Ireland; 7 Health Information and Quality Authority, Dublin 7, Ireland; 8 Department of Pharmacology and Therapeutics, Trinity Health Sciences, The University of Dublin Trinity College, Dublin, Leinster, Ireland

**Keywords:** Altmetric, science communication, media coverage, knowledge dissemination, health research

## Abstract

**Background:**

Scientific publications have been growing exponentially, contributing to an oversaturated information environment. Quantifying a research output’s impact and reach cannot be solely measured by traditional metrics like citation counts as these have a lag time and are largely focused on an academic audience. There is increasing recognition to consider ‘alternative metrics’ or altmetrics to measure more immediate and broader impacts of research. Better understanding of altmetrics can help researchers better navigate evolving information environments and changing appetites for different types of research.

**Objectives:**

Our study aims to: 1) analyse the amount and medium of Altmetric coverage of health research produced by Irish organisations (2017 – 2023), identifying changes over time and 2) investigate differences in the amount of coverage between clinical areas (e.g., nutrition vs. neurology).

**Methods:**

Using Altmetric institutional access, we will gather data on research outputs published 1 January 2017 through 31 December 2023 from active Irish organisations with Research Organisation Registry (ROR) IDs. Outputs will be deduplicated and stratified by their Australian and New Zealand Standard Research Classification relating to ≥1 field of health research: Biological Sciences, Biomedical and Clinical Sciences, Chemical Sciences, Health Sciences, and Psychology. We will clean data using R and perform descriptive analyses, establishing counts and frequencies of coverage by clinical area and medium (e.g., traditional news, X, etc.); data will be plotted on a yearly and quarterly basis where appropriate.

**Results and Conclusions:**

Improved understanding of one’s information environment can help researchers better navigate their local landscapes and identify pathways for more effective communication to the public. All R code will be made available open-source, allowing researchers to adapt it to evaluate their local landscapes.

## Introduction

Scientific publications have grown exponentially in recent years, contributing to an ‘infodemic’ as seen during the Covid-19 pandemic ^1– 3^. With around 1.5 million new items being added to PubMed per year, or 2 papers per minute, there is a need to demonstrate impact of the research to ensure that the scientific community is not favouring quantity over quality ^4^. For many years, impact measures were focused on citation counts which are primarily metrics of influence to other academics working in related fields. However, there have been shifts in the past decade away from traditional bibliometrics like citation counts as they can be poor predictors of quality and impact, have a large lag time, and are largely focused on academics ^5, 6^. Increasingly researchers, institutions, and funders are considering ‘alternative metrics’ or altmetrics to measure the real-time impacts of research on a broader population ^7^.

Traditional bibliometrics and altmetrics are complementary measures which provide a more complete picture of impact ^8^. Altmetric tracks the immediate online attention given to scientific publications ( https://www.altmetric.com/about-us/our-data/how-does-it-work/), making it an invaluable tool in crowded information environments. It provides an ‘Altmetric-Attention Score’ (AAS) which factors in sources from social media (e.g., Facebook, X), YouTube videos, newspapers, policy documents, Wikipedia, question-and-answer sites (e.g., Stack Overflow), and more ( https://www.altmetric.com/about-us/our-data/our-sources/). The broadness of this measure of impact offers the opportunity capture a more diverse picture of the impact of a piece of research ^9^. The AAS has been found to be associated with citation counts ^7, 10^, journal impact factor ^11^, and the likelihood of being cited in policy documents ^12^. The attention score also has showed differences during the Covid-19 pandemic where Covid-19 related work had significantly higher AAS than for non-Covid-19 articles in 2020 ^8^. Despite broad criticism about how the AAS is calculated and its reproducibility, it remains one of the strongest proxies for social attention and is widely used in the health and social sciences ^13^, particularly as it the health sciences often show the highest Altmetric data coverage and attention ^14– 16^. Previous research evaluating coverage of Web of Science documents indexed on Altmetric.com has shown relatively high percentage of coverage for Ireland (68%), especially in comparison to other European countries ^16^.

 How research findings are presented through domestic news can influence behaviour and risk perceptions ^17– 20^. Therefore, it would be beneficial for health researchers and healthcare practitioners to better understand the influence that the dissemination of research publications and their subsequent coverage can have on public behaviour. Analyses of research publication’s online impact can provide insights on effective communication strategies for research outputs produced during COVID-19, for future pandemics, and in generally oversaturated complex information environments ^21^. Despite the shift to online global social media, countries still have their own unique landscape and conditions with varying rates of audience engagement and trust in their local and international news sources ^22^.

According to the 2023 Reuters Digital News report ^22^, Irish consumers have bucked international trends, with levels of trust in news remaining fairly high. Almost half (47%) agreed that they can trust most news most of the time. Ireland can also be considered an outlier in other ways, with 96% of adults having received the full primary COVID-19 vaccination course in 2022, compared to the EU average of 82% ^23, 24^ and registering the fourth lowest rate of excess deaths among OECD countries during the Covid-19 pandemic (2020–2022). While the strong uptake of vaccination clearly had an impact, evidence-based public health messaging (e.g., https://ihealthfacts.ie/) and clear messaging in the mainstream media likely also contributed to beneficial behaviour changes ^25, 26^.

Ireland has also recently made significant investment in health research and healthcare reforms through its Health Service Executive (HSE) Action Plan for Health Research (2019 – 2029) ^27^ and Sláintecare reform (initially launched in 2017) ^28, 29^. The Action Plan emphasizes that dissemination and implementation of research are essential to achieving impactful policy and practices that meet the needs of patients, the health service, and policy makers ^27^. Furthermore, from 2020, the Irish Research e-Library (IReL) signed the first open access publishing agreements, providing researchers with easier access to open access publishing ^30^. Within this context of healthcare and publication reform and the Covid-19 pandemic, we have proposed to include data prior to these changes and the pandemic, to provide some baseline proxy, as well as data throughout and ‘post’-pandemic. This supports our aim to map a piece of the complex local landscape of research in Ireland, using a cross-sectional analysis of Altmetric data (2017 – 2023) and see how it has evolved since before, during, and after the Covid-19 pandemic. A better understanding of the online impact of recent health research can help researchers, and the communication specialists who help disseminate their work, identify pathways for more effective communication to the public. Innovations and dissemination can be improved through better recognition of changing narratives and key players.

## Objectives

Our primary objective is to analyse the amount and type (i.e., medium) of online attention given to health research produced by Irish organisations in recent years (2017 – 2023). We aim to investigate differences over time to identify changing trends, particularly as the online coverage of health research may have been affected during the Covid-19 pandemic. Our secondary objective is to identify differences in the amount of coverage between areas (e.g., nutrition vs. neurology). Our main research questions are: how did research outputs change over time (amount, open access status, clinical area prevalence, etc.) and what are the differences in Altmetric coverage of research outputs during this period? We are also interested in: the relationships between the Altmetric data (as indicate by the Altmetric Attention Score) and citation data

## Methods

This project will be a cross-sectional study as it is a snapshot of online attention given to research outputs (i.e., articles, books, and chapters) from one defined time period (2017 – 2023) with the main focus to provide descriptive prevalence insights and changing trajectories of online attention. Project findings will be reported according to the STROBE Statement ^31^

### Dataset

Data for this study will be gathered from Altmetric institutional access. Altmetric (altmetric.com) tracks 4,000 global news outlets, X (formerly Twitter), YouTube, Reddit, Stack Overflow (Q&A), a curated list of public Facebook pages, blogs, public policy documents, IFI CLAIMS patents, Wikipedia, Mendeley, and Publons. It uses a unique identifier (e.g., DOI, PubMedID, arXiv ID, ISBN, etc.) to track online attention given to a specific research output. ( https://www.altmetric.com/about-us/our-data/how-does-it-work/) Research outputs included are largely journal articles although book chapters and books will also be included. Of note, some items within Altmetric are classified as ‘news’ but can be considered articles as they are perspectives, commentaries, overviews, or hot topics. 

We will use Altmetric Explorer to search for all research outputs published between 1 January 2017 and 31 December 2023 from Irish organisations that have Research Organisation Registry (ROR) IDs. ROR is a global registry of open persistent identifiers for research organisations which helps link researchers and their outputs to institutions across sectors (e.g., education, government, healthcare, non-profit, etc.) ( https://ror.org/about/). Altmetric uses the predecessor system, the Global Research Identifier Database (GRID) ( https://www.grid.ac/) which maps to ROR. As of 9 April 2024, there were 663 research organisations with Ireland listed as their country of address. We searched both active and inactive IDs in case an organisation had outputs during the time period but then decided to inactivate their indexing GRID (e.g., they published in 2017–2019 but then deactivated their ID). The lead author (MKS) and two medical students will use the Altmetric Explorer interface to search for and download research outputs published within our date frame from each individual organisation. Datasets will be tracked using a tracking log in Excel to record the downloader (e.g., MKS), total number of research outputs, number of outputs mentioned, filename, and download date. If an organisation has a least 1 output, we will download their data as a csv file using a standard naming notation (ID_YYYY-MM-DD). These csv files will be stored in one folder, spot checked for completeness (MKS), then combined into one dataset which will include research output data from all organisations producing output from 2017 – 2023. Of note, the datasets have the same 46 variables, will be downloaded in UTF-8 to account for non-English characters, and dates will be checked prior to stacking. This tracking log and our RMarkdown code detailing combining of datasets, cleaning, pre-processing, and more can be available on our Open Science Framework accompanying our results manuscript ^32^. While the Altmetric API ( https://www.altmetric.com/solutions/altmetric-api/) is also available for information retrieval, we did not find it suitable for pulling data based on an organisation’s GRID ID. 

Each dataset (e.g., csv file) contains 46 standard columns or variables, one of which is the field of research. Each research output in the dataset is classified to at least one field of research (FoR) using the 2020 Australian and New Zealand Standard Research Classification (ANZSRC) system. The ANZSRC is a hierarchical system which contains divisions (broad subject areas or research disciplines) which are further detailed into subsets: groups and fields. ANZSCR was developed for use in the measurement and analysis of research and experimental development (R&D) statistics in Australia and New Zealand ^33^. In instances where there is a lack of information and it cannot be classified at this level, the code is assigned based on a journal-level classification.

For the purposes of our project we are primarily interested in the following Divisions of biomedical research listed in Table 1: Biological Sciences (31), Biomedical and Clinical Sciences (32), Chemical Sciences (34), Health Sciences (42), and Psychology (52). Excluded areas include: Agricultural, Veterinary and Food Sciences (30), Built Environment and Design (33), Commerce, Management, Tourism and Services (35), Creative Arts and Writing (36), Earth Sciences (37), Education (39), Engineering (40), Environmental Sciences (41), History, Heritage and Archaeology (43), Human Society (44), Indigenous Studies (45), Information and Computing Sciences (46), Language, Communication and Culture (46), Law and Legal Studies (48), Mathematical Sciences (49), Philosophy and Religious Studies (50), and Physical Sciences (51). As research outputs can be classified to several areas, as long as at least one of our included Divisions is in the field, the output will be included. For example, if a study is about the health impacts of environmental pollution and contamination, it could be classified under Biomedical and Clinical Sciences (32) and Environmental Sciences (41), thus it would still be retained.

**Table 1.** Included divisions of research according to the Australian and New Zealand Standard Research Classification ^33^.

Included Divisions	31 BIOLOGICAL SCIENCES	32 BIOMEDICAL AND CLINICAL SCIENCES	34 CHEMICAL SCIENCES	42 HEALTH SCIENCES	52 PSYCHOLOGY
Included Groups	3101 Biochemistry and cell biology 3102 Bioinformatics and computational biology 3103 Ecology 3104 Evolutionary biology 3105 Genetics 3106 Industrial biotechnology 3107 Microbiology 3108 Plant biology 3109 Zoology 3199 Other biological sciences	3201 Cardiovascular medicine and haematology 3202 Clinical sciences 3203 Dentistry 3204 Immunology 3205 Medical biochemistry and metabolomics 3206 Medical biotechnology 3207 Medical microbiology 3208 Medical physiology 3209 Neurosciences 3210 Nutrition and dietetics 3211 Oncology and carcinogenesis 3212 Ophthalmology and optometry 3213 Paediatrics 3214 Pharmacology and pharmaceutical sciences 3215 Reproductive medicine 3299 Other biomedical and clinical sciences	3401 Analytical chemistry 3402 Inorganic chemistry 3403 Macromolecular and materials chemistry 3404 Medicinal and biomolecular chemistry 3405 Organic chemistry 3406 Physical chemistry 3407 Theoretical and computational chemistry 3499 Other chemical sciences	4201 Allied health and rehabilitation science 4202 Epidemiology 4203 Health services and systems 4204 Midwifery 4205 Nursing 4206 Public health 4207 Sports science and exercise 4208 Traditional, complementary and integrative medicine 4299 Other health sciences	5201 Applied and developmental psychology 5202 Biological psychology 5203 Clinical and health psychology 5204 Cognitive and computational psychology 5205 Social and personality psychology 5299 Other

**Figure 1.**
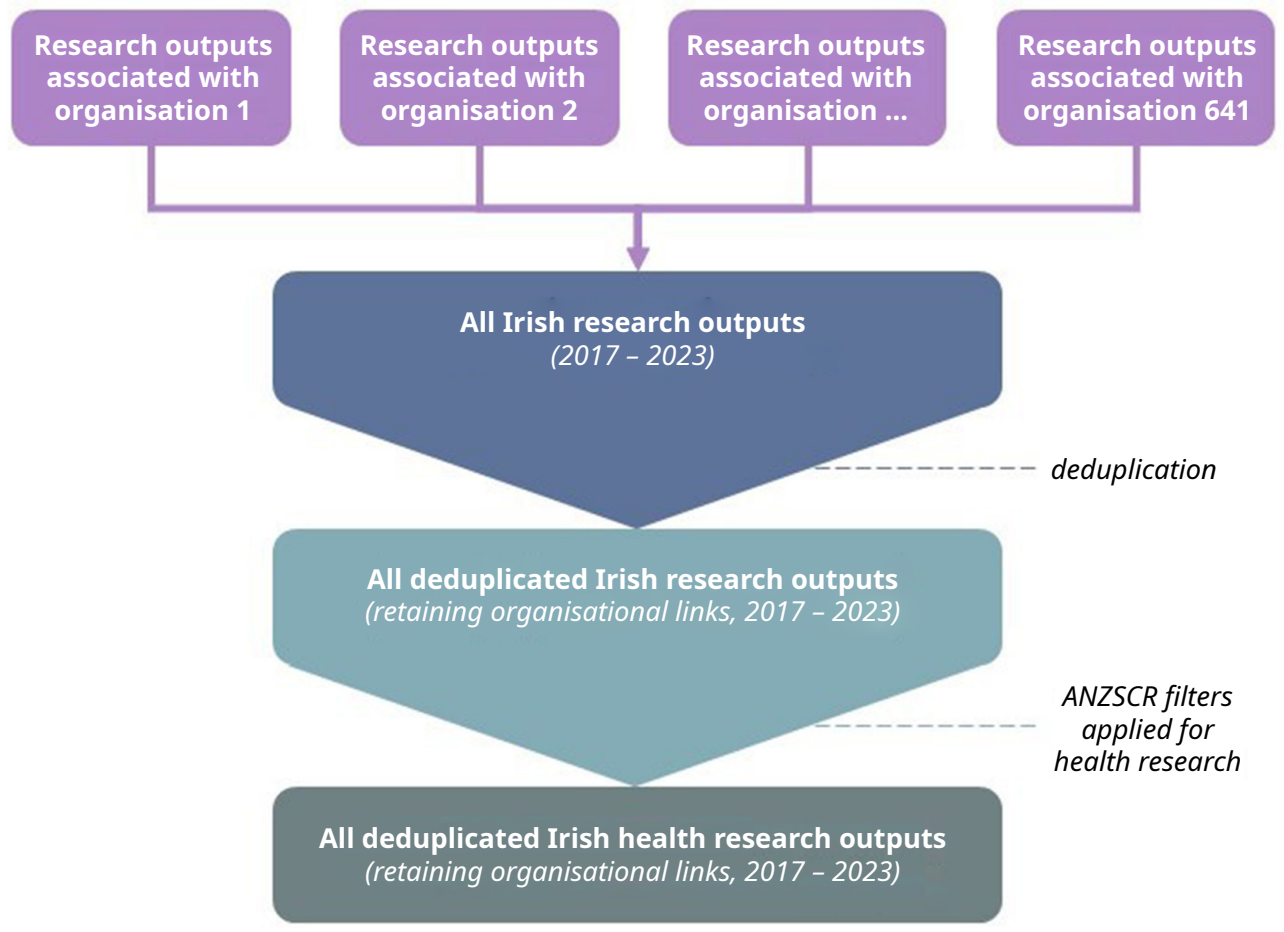
Flow diagram of dataset creation.

All individual organisation datasets will be stacked and combined, deduplicated, and filtered to only contain research outputs pertaining to at least one field of health research as defined by the ANZSRC ( Figure 1). Deduplication will be based on an output’s DOI and impact metrics should be identical across outputs with the same DOI. Of note, author affiliations with a research output do not depend on placement (e.g., first, corresponding). We will create a new variable to maintain links in the likely case where a research output is associated with multiple organisations. For example, if research output X was published by both author 1 at organisation A and author 2 at organisation B, the research output should contain the same DOI which is the information that is being used to track all the attention, therefore, that information should not change and deduplication does not pose a threat to data loss.

### Analysis

We will use R version 4.3.2 ( https://shiny.rstudio.com/) to analyse data for this project. As data will be gathered using an institutional license for Altmetric Explorer, we will use R Markdown ( https://rmarkdown.rstudio.com/) to create an html document to maintain privacy of the proprietary data but promote reproducible research practices. The .rmd file will also be available on GitHub ( https://github.com/sharpmel) and the project will be registered on the Open Science Framework. ( https://osf.io/kfct6/)

Data will be cleaned using R and descriptive analyses will be performed for general bibliometric information such as: the type of research output (i.e., article, book, chapter); open access status and type; 20 most frequent journals, funders, and organisations in our dataset; the prevalence of sectors (e.g., education, healthcare); and the five subject areas of health research and their subdivisions (yearly). To investigate trends over time, counts of frequencies will be plotted on a quarterly and yearly basis for the overall number of research outputs and yearly for subject and subdivision areas, sectors, and publishers.

For Altmetric analyses, we will report counts, and averages and medians per medium (e.g., X, Facebook, policy documents) for both the deduplicated dataset as a whole and for the deduplicated dataset with the zero-count AAS removed as there may be large amounts of zero counts in our dataset, potentially skewing measures of central tendency. We will also report the number of outputs with a score of 20 or above as Altmetric has indicated that this is a general score which can be considered as doing better than most of its ‘colleagues’ ^34^. All medium data will be plotted on a quarterly and yearly basis from 1 January 2017 through 31 December 2023. We will also segment data by subject areas and divisions. As the World Health Organisation (WHO) declared Covid-19 a pandemic on 11 March 2020 ^35^ and no longer a public health emergency of international concern on 5 May 2023 ^36^, these cut points will be used for general discussions on pre-, during, and post-pandemic. 

Assuming an adequately sized dataset, we will also investigate whether Altmetric Attention Scores correlate to traditional article-level citation metrics. Crossref lags significantly behind other major sources in terms of comprehensiveness ^37^, therefore we will use OpenAlex to obtain citation data. OpenAlex ^38, 39^ is an open-source catalogue which includes metadata for 209 million outputs like journal articles and books and its average reference numbers are comparable to Web of Science and Scopus ^40^. We will use zero-inflated negative binomial regression to account for the large number of AAS scores of zero. As articles in 2017 have had more time to accrue citations than those published in 2023, we will restrict our analyses and split our dataset by year. Previous research has indicated that a 3-year citation window is relatively stable so we will perform these analyses on the data from 2017–2020 only ^41, 42^. 

***Field and clinical area.*** Although the FoR classification system will give some insights as to content areas which are being covered, it does not provide more granular detail. OpenAlex provides a more thorough and in-depth view of academic research as it contains 4,500 topics and builds upon and fills the gap from the discontinued Microsoft Academic Graph (MAG) ^43, 44^. We will gather topic information by using the OpenAlexAPI to pull this information matched by the DOIs in our final dataset. OpenAlex uses a hierarchical system that organises topics into levels ranging from broad to more specific, from domain to field, subfield, and finally topic system. We will focus on lower level topics and will report the 20 most frequent topics per year.

***Limitations.*** Our main limitation is the dataset itself and the quantitative focus of measuring impact. Firstly, Altmetric does not track certain platforms such as LinkedIn, TikTok, and Instagram, thus its generalisability is diminished. It has also been reported to have issues tracking publications with multiple versions (i.e., a pre- and post-print) and the replication of the data can sometimes be difficult due to constantly changing access agreements with data providers ^45^. 

The lack of a links between preprints (manuscripts uploaded to databases without peer review) and postprints is a larger issue (peer reviewed journal articles) within academic publishing ^46^ as each item is assigned a unique DOI and there are challenges indexing preprints alongside their peer-reviewed publication. Of note, in the Altmetric dataset, preprints are included and tracked, they just have their own individual altmetrics separate from the final publication. Preprints played an unprecedented role in disseminating Covid-19 research ^47^, so we will include them in our dataset, account for this in statistical analyses, and explicitly disclose this preprint prevalence in our dataset, and frame our findings with this in mind. 

Furthermore, although higher scores can be expected from newer papers as time since publication has shown to be associated with Altmetric scores ^11^, even running searches one month apart resulted in changing numbers as old articles can be brought up for discussion at any time. Altmetric data has also been noted to be prone to manipulation and artificial inflation ^48^ and some sources are particularly unstable, with certain items ‘vanishing’ ^13^. We will try to address this by pulling the data in a discrete period of time and we have included a time buffer (i.e., the end of 2023). However, we do recognise that certain mediums may still be ‘incomplete’ as they have different trajectories of attention growth – for example, Twitter (X) attention starts and ends quickly, Mendeley readers accumulate quickly but continue to grow over the years, and policy attention is the slowest form of impact to accumulate ^49^.

The quantitative focus of the metrics in the dataset can also lose the context or tone of the coverage and does not account for ‘dose’, i.e., where a mention may be an extensive discussion or extremely brief. A high AAS does not indicate a high-quality piece of research ^50^ and the AAS is a measure of attention, not quality. However, recent work comparing altmetrics to norm-referenced peer review scores from the UK Research Excellence Framework 2021 found that Altmetric correlated more strongly with research quality than previously thought although there is large variability with the strength of correlations amongst mediums and between fields (e.g., stronger in health and physical sciences than in the arts and humanities) ^14^.

Providing a broad overview of the online attention given to health research in Ireland in recent years is our primary objective. Our datasets are not meant for social listening purposes and audience metrics may be limited. However, our project’s results may provide a basis to build upon for future studies investigating *how* the media is actually covering the work. Altmetric may also be a flawed metric of impact on the public as previous research has shown that most tweets came from within academia with other academics interacting with them ^51^. Notably, the data in our project likely will contain more health research produced from the academic sector as they primarily communicate via articles, books, and chapters, however, we have included pharmaceutical agencies, governmental health bodies, and hospitals (where they have an ROR) which may provide a broader overview of the online coverage of health research in Ireland. A recent bibliometric analysis of HRB supported publications ^52^ found the academic sector well-represented although our project is much broader in its scope.

## Discussion and implications

A better understanding of the amount and type of online attention given to health research involving Irish organisations can identify changing trends and gaps in attention. By looking at organisation-linked data and unique research outputs, we can provide insights to researchers and organisations (particularly universities) looking to evaluate the impact of their work and identify the strengths and weaknesses of their research portfolios. Results may be particularly useful for researchers and communication specialists who are aiming disseminate their research to the public and find ‘airtime’ in a particularly noisy information environment. Our use of open source coding also will offer a reproducible workflow for future monitoring and further investigations into the content of the health research coverage itself. Results from our ‘case study’ focused on the Irish landscape can also be used to compare and contrast with other countries. The time period chosen for our project may be of particular interest due to the variation in governmental responses to Covid-19 and differing rates of personal protective behaviours (e.g., vaccinations, masking, etc.). Overall, the project should provide us with a piece of the puzzle of the landscape of online attention given to health research in Ireland.

## Data Availability

No data are associated with this article.
