# Supplementary material for: Altmetric coverage of health research in Ireland 2017-2023: a protocol for a cross-sectional analysis
Source: HRB Open Res. 2024 Oct 22;7:36. Originally published 2024 Jun 18. [Version 3] doi: 10.12688/hrbopenres.13895.3 (PMC11443184; doi:10.12688/hrbopenres.13895.3)
Supplement: Peer review history

# Reviewer report: Reviewer response for version 3

**DOI:** 10.21956/hrbopenres.15381.r42908

**Published:** 2024-11-04

**Authors:** Andreas Nishikawa-Pacher

**Affiliations:** 1 Vienna School of International Studies and University of Vienna, Vienna, Austria; 2 Bibliothek, TU Wien, Vienna, Austria

The minor issue about the potential confusion between 'concepts' and 'topics' in OpenAlex has been resolved.

Is the study design appropriate for the research question?

Yes

Is the rationale for, and objectives of, the study clearly described?

Yes

Are sufficient details of the methods provided to allow replication by others?

Yes

Are the datasets clearly presented in a useable and accessible format?

Not applicable

Reviewer Expertise:

bibliometrics

I confirm that I have read this submission and believe that I have an appropriate level of expertise to confirm that it is of an acceptable scientific standard.

# Reviewer report: Reviewer response for version 2

**DOI:** 10.21956/hrbopenres.15326.r42289

**Published:** 2024-09-30

**Authors:** Nicole Llewellyn

**Affiliations:** 1 Georgia Clinical and Translational Science Alliance, Emory University, Georgia, USA

This article presents a protocol for an investigation of Altmetric attention to health related research conducted at Irish institutions from 2017-2023. The introduction makes solid arguments for examining Altmetrics, which is a novel area of publication analysis that itself needs more attention in the literature. The methodology is thorough and well-explained. Suggest describing what STROBE is, adding the caveat that an AAS of 20+ was defined before COVID and may not apply the same way now, and suggest adding the relevant reference to Llewellyn & Nehl, 2022 [Ref 1] regarding AAS and adjusted citation scores.

Is the study design appropriate for the research question?

Yes

Is the rationale for, and objectives of, the study clearly described?

Yes

Are sufficient details of the methods provided to allow replication by others?

Yes

Are the datasets clearly presented in a useable and accessible format?

Not applicable

Reviewer Expertise:

Evalution, health publication analysis

I confirm that I have read this submission and believe that I have an appropriate level of expertise to confirm that it is of an acceptable scientific standard.

## References

[1] : Predicting citation impact from altmetric attention in clinical and translational research: Do big splashes lead to ripple effects?. *Clin Transl Sci* .2022;15(6) : 10.1111/cts.13251 1387-1392 10.1111/cts.13251 35146918 PMC9199871

# Reviewer report: Reviewer response for version 2

**DOI:** 10.21956/hrbopenres.15326.r42245

**Published:** 2024-09-19

**Authors:** Andreas Nishikawa-Pacher

**Affiliations:** 1 Vienna School of International Studies and University of Vienna, Vienna, Austria; 2 Bibliothek, TU Wien, Vienna, Austria

The authors provided thoughtful responses, and the revised manuscript does seem to convey a more feasible approach.

 I have only one comment left regarding the OpenAlex-based "topics". I am not sure whether the authors may have mistaken, in parts, the OpenAlex-based "topics" with the OpenAlex-based "concepts", the latter of which has been deprecated (if I remember correctly). I can understand that one would mistake them given that they have a similar function, and given that the "topics" have only recently replaced the "concepts" in OpenAlex.

 The (outdated) "concepts" used to have levels (like level 1, level 2), and perhaps keywords like the ones cited by the authors ("anxiety", "telemedicine").

 In contrast, the (newer) "topics" do not have "levels" anymore (but a "topic" is part of a "subfield" which, in turn, is part of a "field" which, in turn, is part of a "domain").

 In addition, the "topics" are more concrete. For example, instead of a concept called "anxiety", there are three topics that are named as such:

 1.) "Cognitive Mechanisms of Anxiety and Depression"

 2.) "The Relationship Between Music and Anxiety Management"

 3.) "Dental Anxiety and Anesthetic Management in Dentistry"

 Here is an API-link to the example of "Dental Anxiety ...": https://openalex.org/topics/T12145

 The authors certainly looked at "topics" correctly (for instance, ref. 44 leads to a Google Sheets page enumerating all the "topics"), but I think that the paragraph they inserted on "topics" also contained, perhaps erroneously, some references to the outdated "concepts".

 This is not a huge matter, but I thought I'd point it out in case it may be helpful.

 Other than that, the draft seems very much enhanced!

Is the study design appropriate for the research question?

Yes

Is the rationale for, and objectives of, the study clearly described?

Yes

Are sufficient details of the methods provided to allow replication by others?

Yes

Are the datasets clearly presented in a useable and accessible format?

Not applicable

Reviewer Expertise:

bibliometrics

I confirm that I have read this submission and believe that I have an appropriate level of expertise to confirm that it is of an acceptable scientific standard, however I have significant reservations, as outlined above.

# Response

**Published:** 2024-10-13

**Authors:** Melissa Sharp

**Affiliations:** Royal College of Surgeons in Ireland, Ireland

Thank you for looking at our new version and for your thoughtful comments and feedback. We argee that the text in our second version may have caused confusion between concepts and topics. As you noted, due to the deprecation of concepts, we will use topics. We have edited the section accordingly.

# Reviewer report: Reviewer response for version 1

**DOI:** 10.21956/hrbopenres.15238.r40895

**Published:** 2024-07-19

**Authors:** Andreas Nishikawa-Pacher

**Affiliations:** 1 Vienna School of International Studies and University of Vienna, Vienna, Austria; 2 Bibliothek, TU Wien, Vienna, Austria

Thank you for allowing me to review this study protocol which eyes to study the altmetric coverage of health research produced by Irish organisations, including with disaggregations regarding specific clinical areas and study types.

 While I am no expert in the methodical approach as such (for instance, I cannot comment on the soundness of the use of latent Dirichlet allocation in this particular context), from the viewpoint of someone who engages in scientometric studies, the study protocol seems to be fine and worthwhile to be undertaken.

 The reasons given for utility and the prospects of the study are well-argued. As regards the methods, the data sources should be good to use. From my experience, it should be possible to combine ROR/GRID with the Altmetric API without too many data losses (though there will certainly be a few omissions when affiliation names are not stored correctly in the publishers' metadata).

 One aspect that I am uncertain about is whether the topic modelling approach for obtaining the research field and clinical area is really that helpful, especially since the topic modelling eyed would only look at the title of each publication. It would be interesting to find out whether the "topics" that are available as open data from OpenAlex could likewise serve as a good, if not better, equivalent regarding the clinical area, since OpenAlex' "topic"-assignment is based not only on the title, but also on the abstract, the journal/source, and the references of each respective paper (see: https://docs.openalex.org/api-entities/topics).

 With regards to the classification of study design, the approach sounds innovative; but I am also unsure about the success their approach will yield. It is certainly worthwhile to try it out, and it would be interesting to see the result. (And just as a sidenote, I wonder whether there is a good theoretical reason to believe that some study designs will structurally have a higher Altmetric Attention Score. For instance, would papers with 'randomized controlled trial' in the title or in the keywords get more attention in mass media because journalists may think that such studies are more reliable than others? Why would that be? Would a 'systematic review' catch more attention than a 'scoping review'? This is not to say that I'd demand from the authors that they come up with convincing theories; it is just an intriguing idea that was stimulated by the authors' study protocol.)

 When it comes to the Discussion section, one future study that might be interesting would be to speculate whether a comparison between Ireland and another countries (e.g., one which, in the aggregate, was more sceptical regarding the COVID-19 vaccinations) would yield different results (given the reasons stated in the Introduction for the choice of research object).

 Just as minor remark, I think the second paragraph should speak of an "Altmetric Attention Score" (rather than Altmetric-mentioned score).

 Finally, I agree with the other peer-reviewer (Dimitry Stephan) that high Altmetric Attention Scores do not mean that a paper is really impactful & of a high quality. In fact, there are studies suggesting that high Altmetric Attention Scores can serve as a predictor of a retraction [Ref -1]. And there are enough "funny" or "strange" papers that lead to almost viral discussions in social media precisely because of their errors and flaws.

 Anyway, the study protocol is well thought out and promises interesting results. I wish the authors all the best for their undertaking. The only change I would like to see as a reviewer is an explicit discussion on OpenAlex' "topics"; other than that, the study protocol seems perfect from me (notwithstanding my inability to judge on the exact statistical topic modelling method).

Is the study design appropriate for the research question?

Yes

Is the rationale for, and objectives of, the study clearly described?

Yes

Are sufficient details of the methods provided to allow replication by others?

Yes

Are the datasets clearly presented in a useable and accessible format?

Not applicable

Reviewer Expertise:

bibliometrics

I confirm that I have read this submission and believe that I have an appropriate level of expertise to confirm that it is of an acceptable scientific standard, however I have significant reservations, as outlined above.

## References

[1] : Media and social media attention to retracted articles according to Altmetric. *PLoS One* .2021;16(5) : 10.1371/journal.pone.0248625 e0248625 10.1371/journal.pone.0248625 33979339 PMC8115781

# Response

**Published:** 2024-08-19

**Authors:** Melissa Sharp

**Affiliations:** Royal College of Surgeons in Ireland, Ireland

Thank you for allowing me to review this study protocol which eyes to study the altmetric coverage of health research produced by Irish organisations, including with disaggregations regarding specific clinical areas and study types.

 While I am no expert in the methodical approach as such (for instance, I cannot comment on the soundness of the use of latent Dirichlet allocation in this particular context), from the viewpoint of someone who engages in scientometric studies, the study protocol seems to be fine and worthwhile to be undertaken.

 The reasons given for utility and the prospects of the study are well-argued. As regards the methods, the data sources should be good to use. From my experience, it should be possible to combine ROR/GRID with the Altmetric API without too many data losses (though there will certainly be a few omissions when affiliation names are not stored correctly in the publishers' metadata).

**Response: **Thank you for the time spent reviewing our manuscript and for providing thoughtful and thorough feedback! The comment regarding the quality of the metadata is an important one (17). We will perform spot-checking, combining data sources and monitoring any retrieval issues, and make our R code open source to promote reproducibility and transparent reporting.

 One aspect that I am uncertain about is whether the topic modelling approach for obtaining the research field and clinical area is really that helpful, especially since the topic modelling eyed would only look at the title of each publication. It would be interesting to find out whether the "topics" that are available as open data from OpenAlex could likewise serve as a good, if not better, equivalent regarding the clinical area, since OpenAlex' "topic"-assignment is based not only on the title, but also on the abstract, the journal/source, and the references of each respective paper (see: https://docs.openalex.org/api-entities/topics).

**Response: **Thank you for the suggestion of OpenAlex, we have added a section to the methods section to pivot to this approach: *“OpenAlex  provides a more thorough and in-depth view of academic research as it contains 65,000 Wikidata concepts based on Microsoft Academic Graph (MAG)*(9) *and enhanced with machine learning, natural language processing, citation analysis, an expert feedback. We will gather the topic-, mid-, and lower- level concepts and subfield information by using the OpenAlexAPI to pull this information matched by the DOIs in our final dataset. Mid- and lower-level concepts could include things like ‘pediatric oncology’, ‘telemedicine’, or ‘anxiety.’ We will report the top 20 terms per year and if possible visualise this information using the VOS-Viewer free software* *. *(11) *”*

 With regards to the classification of study design, the approach sounds innovative; but I am also unsure about the success their approach will yield. It is certainly worthwhile to try it out, and it would be interesting to see the result. (And just as a sidenote, I wonder whether there is a good theoretical reason to believe that some study designs will structurally have a higher Altmetric Attention Score. For instance, would papers with 'randomized controlled trial' in the title or in the keywords get more attention in mass media because journalists may think that such studies are more reliable than others? Why would that be? Would a 'systematic review' catch more attention than a 'scoping review'? This is not to say that I'd demand from the authors that they come up with convincing theories; it is just an intriguing idea that was stimulated by the authors' study protocol.)

**Response: **Thank you for this note. Anecdotally we have heard from colleagues in pre-clinical and evidence synthesis work that it is harder to get public interest and engagement in their work. This led us to the initial proposal. However, due to the final size of our dataset we no longer thought it was feasible to conduct the study design exploration aspect of our project and have taken this information out of our protocol (both study objectives and the study design section. However, we will still be creating the dataset which perhaps can be used to explore this in the future.

 When it comes to the Discussion section, one future study that might be interesting would be to speculate whether a comparison between Ireland and another countries (e.g., one which, in the aggregate, was more sceptical regarding the COVID-19 vaccinations) would yield different results (given the reasons stated in the Introduction for the choice of research object).

**Response: **This is an important note. We have added related text to the discussion and implications section: *“Results from our ‘case study’ focused on the Irish landscape can also be used to compare and contrast with other countries. The time period chosen for our project may be of particular interest due to the variation in governmental responses to Covid-19 and differing rates of personal protective behaviours (e.g., vaccinations, masking, etc.).”*

 Just as minor remark, I think the second paragraph should speak of an "Altmetric Attention Score" (rather than Altmetric-mentioned score).

**Response: **We have fixed this typo.

 Finally, I agree with the other peer-reviewer (Dimitry Stephan) that high Altmetric Attention Scores do not mean that a paper is really impactful & of a high quality. In fact, there are studies suggesting that high Altmetric Attention Scores can serve as a predictor of a retraction [Ref -1]. And there are enough "funny" or "strange" papers that lead to almost viral discussions in social media precisely because of their errors and flaws.

**Response: **We agree that a high AAS should not be synonymous with good or indicate a high-quality piece of research. We have added text relating to this to the limitations section: “A high AAS does not indicate a high-quality piece of research(26) and the AAS is a measure of attention, not quality. However, recent work comparing altmetrics to norm-referenced peer review scores from the UK Research Excellence Framework 2021 found that Altmerics correlated more strongly *with research quality than previously thought although there is large variability with the strength of correlations amongst mediums and between fields (e.g., stronger in health and physical sciences than in the arts and humanities).* *(3)* *”*

 Anyway, the study protocol is well thought out and promises interesting results. I wish the authors all the best for their undertaking. The only change I would like to see as a reviewer is an explicit discussion on OpenAlex' "topics"; other than that, the study protocol seems perfect from me (notwithstanding my inability to judge on the exact statistical topic modelling method).

**Response: **Per our earlier response, we have pivoted our approach and removed the LDA topic modelling. Thank you for your thoughtful and positive comments.

# Reviewer report: Reviewer response for version 1

**DOI:** 10.21956/hrbopenres.15238.r40898

**Published:** 2024-07-16

**Authors:** Fei Yu

**Affiliations:** 1 University of North Carolina at Chapel Hill, North Carolina, USA

The manuscript analyzes the online attention of health research outputs produced by Irish organizations from 2017 to 2023 using Altmetric data. It emphasizes using altmetrics as an alternative measure to address the limitations and biases of traditional bibliometric measures, such as raw citation counts. However, several areas could benefit from further clarification and improvement.

**Areas for Improvement:**

**1. Literature Review:** The introduction could include a more extensive review of relevant literature on applying altmetrics to assess online attention (digital impact) in the field of biomedical and health sciences. A simple search in PubMed will yield many relevant studies. This would provide a stronger foundation for the study's rationale and methodology.

**2. Research Questions:** Although the authors state their primary and secondary objectives, no specific research questions are proposed to guide the data search and analysis.

**3. Methodological Details:**

- It would be helpful to include more details on the data cleaning and preprocessing steps, particularly regarding how duplicates will be handled and how the dataset will be validated. For example, will the dataset be manually screened by two reviewers independently to ensure its validity and relevance?
- The manuscript should provide a rationale for the selected time frame (2017-2023) and explain any potential biases this period may introduce. This time range covers before, during, and after COVID-19. The authors need to explain what potential changes they expect to observe and why the changes are important.
- If Table 1 is based on the Altmetric help page of "field of research," the authors need to cite the source.
- Since there are no specific research questions or theories/models to guide the data extraction, the authors need to explain the topic mapping using titles. There are existing bibliometric tools for topic mapping (e.g., VOSviewer, Biblioshiny). The authors need to explain why they chose to use titles only rather than abstracts, author keywords, or MeSH terms.
- Additionally, the Altmetric platform supports PubMed search queries. The authors might want to explain why they prefer API for data retrieval instead of the user interface.
- Altmetric.com recommends using an Altmetric attention score of 20 as a benchmark. The authors did not mention this for quantitative analysis.

**4. Limitations:** The manuscript could benefit from addressing potential biases in Altmetric data collection and how these might affect the study's findings.

**Specific Comments:**

- The statement "With around 1.5 million new items being added to PubMed per year, or 2 papers per minute..." could include a reference to the source of this statistic for credibility.
- The sentence "Ireland can also be consider an outlier in other ways..." has a grammatical error ("consider" should be "considered").

Overall, the manuscript presents a potentially valuable contribution to understanding online attention to health research in Ireland. However, the research protocol needs improvement, particularly in the areas of method design and justification.

Is the study design appropriate for the research question?

Partly

Is the rationale for, and objectives of, the study clearly described?

Yes

Are sufficient details of the methods provided to allow replication by others?

Partly

Are the datasets clearly presented in a useable and accessible format?

No

Reviewer Expertise:

Health sciences research, bibliometrics

I confirm that I have read this submission and believe that I have an appropriate level of expertise to state that I do not consider it to be of an acceptable scientific standard, for reasons outlined above.

# Response

**Published:** 2024-08-19

**Authors:** Melissa Sharp

**Affiliations:** Royal College of Surgeons in Ireland, Ireland

The manuscript analyzes the online attention of health research outputs produced by Irish organizations from 2017 to 2023 using Altmetric data. It emphasizes using altmetrics as an alternative measure to address the limitations and biases of traditional bibliometric measures, such as raw citation counts. However, several areas could benefit from further clarification and improvement.

**Response:** Thank you for the time spent reviewing our protocol and for the thoughtful comments, questions, and suggestions.

 Areas for Improvement:

 1. Literature Review: The introduction could include a more extensive review of relevant literature on applying altmetrics to assess online attention (digital impact) in the field of biomedical and health sciences. A simple search in PubMed will yield many relevant studies. This would provide a stronger foundation for the study's rationale and methodology.

**Response: **We have edited and added additional information about the value of altmetrics/Altmetric as a measure of alternative impact of research to the paragraph describing Altmetric: *“The AAS has been found to be associated with citation counts,* *7* *,* *9* *journal impact factor* *10* *, and the likelihood of being cited in policy documents.* *11* *The attention score also has showed differences during the Covid-19 pandemic where Covid-19 related work had significantly higher AAS than for non-Covid-19 articles in 2020. *(1) *Despite broad criticism about how the AAS is calculated and its reproducibility, it remains one of the strongest proxies for social attention and is widely used in the health and social sciences *(2) *, particularly as it the health sciences often show the highest Altmetric data coverage and attention. *(3–5) *Previous research evaluating coverage of Web of Science documents indexed on Altmetric.com has shown relatively high percentage of coverage for Ireland (68%), especially in comparison to other European countries. *(5) *”*

 2. Research Questions: Although the authors state their primary and secondary objectives, no specific research questions are proposed to guide the data search and analysis.

**Response: **We have clarified our research questions in the objectives section and have removed part of our secondary objective (relating to study types): *“*Our main research questions are: how did research outputs change over time (amount, open access status, clinical area prevalence, etc.) and what are the differences in Altmetric coverage of research outputs during this period? We are also interested in: how are the relationships between the Altmetric data (as indicate by the Altmetric Attention Score) and citation data?”

 3. Methodological Details:

 It would be helpful to include more details on the data cleaning and preprocessing steps, particularly regarding how duplicates will be handled and how the dataset will be validated. For example, will the dataset be manually screened by two reviewers independently to ensure its validity and relevance?

**Response: **Thank you for this suggestion. We have clarified the information regarding duplicates and data processing and validation: “ *We will use Altmetric Explorer to search for all research outputs published between 1 January 2017 and 31 December 2023 from Irish organisations that have Research Organisation Registry (ROR) IDs. ROR is a global registry of open persistent identifiers for research organisations which helps link researchers and their outputs to institutions across sectors (e.g., education, government, healthcare, non-profit, etc.) (* *https://ror.org/about/* *). Altmetric uses the predecessor system, the Global Research Identifier Database (GRID) (* *https://www.grid.ac/* *) which maps to ROR. As of 9 April 2024, there were 663 research organisations with Ireland listed as their country of address. We searched both active and inactive IDs in case an organisation had outputs during the time period but then decided to inactivate their indexing GRID. (e.g., they published in 2017-2019 but then deactivated their ID). The lead author (MKS) and two medical students will use the Altmetric Explorer interface to search for and download research outputs published within our date frame from each individual organisation. Datasets will be tracked using a tracking log in Excel to record the downloader (e.g., MKS), total number of research outputs, number of outputs mentioned, filename, and download date. If an organisation has a least 1 output, we will download their data as a csv file using a standard naming notation (ID_YYYY-MM-DD). These csv files will be stored in one folder, spot checked for completeness (MKS), then combined into one dataset which will include research output data from all organisations producing output from 2017 - 2023. Of note, the datasets have the same 46 variables, will be downloaded in UTF-8 to account for non-English characters, and dates will be checked prior to stacking. This tracking log and our RMarkdown code detailing combining of datasets, cleaning, pre-processing, and more can be available on our Open Science Framework accompanying our results manuscript.*(6) *While the Altmetric API ( https://www.altmetric.com/solutions/altmetric-api/) is also available for information retrieval, we did not find it suitable for pulling data based on an organisation’s GRID ID.”*

 The manuscript should provide a rationale for the selected time frame (2017-2023) and explain any potential biases this period may introduce. This time range covers before, during, and after COVID-19. The authors need to explain what potential changes they expect to observe and why the changes are important.

**Response:** We have added additional information regarding potential changes expected into the research questions in the objectives section which is provided in further detail in a later response. We have clarified and added information to the last paragraph of the introduction regarding the choice of the time frame and *“Ireland has also recently made significant investment in health research and healthcare reforms through its Health Service Executive (HSE) Action Plan for Health Research (2019 – 2029)* *^23^* *and Sláintecare reform (initially launched in 2017) *(7) *^24^* *.” …*

*“Furthermore, from 2020, the Irish Research e-Library (IReL) signed the first open access publishing agreements, providing researchers with easier access to open access publishing.*(8) *Within this context of healthcare and publication reform and the Covid-19 pandemic, we have proposed to include data prior to these changes and the pandemic, to provide some baseline proxy, as well as data throughout and ‘post’-pandemic. This supports our aim to map a piece of the complex local landscape of research in Ireland, using a cross-sectional analysis of Altmetric data (2017 – 2023) and see how it has evolved since before, during, and after the Covid-19 pandemic. A better understanding of the online impact of recent health research can help researchers, and the communication specialists who help disseminate their work, identify pathways for more effective communication to the public…”*

 If Table 1 is based on the Altmetric help page of "field of research," the authors need to cite the source.

 Response: We have added the reference to the ANZSCR system.

 Since there are no specific research questions or theories/models to guide the data extraction, the authors need to explain the topic mapping using titles. There are existing bibliometric tools for topic mapping (e.g., VOSviewer, Biblioshiny). The authors need to explain why they chose to use titles only rather than abstracts, author keywords, or MeSH terms.

**Response: **We have clarified our research question in a previous response and have provided further details on our planned descriptive analyses which includes the ANZSCR areas: *“Data will be cleaned using R and descriptive analyses will be performed for general bibliometric information such as: the type of research output (i.e., article, book, chapter); open access status and type; top 20 journals, funders, and organisations in our dataset; the prevalence of sectors (e.g., education, healthcare); and the five subject areas of health research and their subdivisions (yearly). To investigate trends over time, counts of frequencies will be plotted on a quarterly and yearly basis for: the overall number of research outputs and yearly for subject and subdivision areas, sectors, and publishers.”*

 Following a suggestion from another reviewer regarding the OpenAlex API and its classification system of topics (developed over many years by experts in the field), we have decided to no longer use our initially proposed topic modelling approach and the Crossref API. We have added text regarding this and will explore using VOSviewer to visualise this (although there may be issues due to file formats and the complexity of our dataset): *“OpenAlex provides a more thorough and in-depth view of academic research as it contains 65,000 Wikidata concepts based on Microsoft Academic Graph (MAG)*(9) *and enhanced with machine learning, natural language processing, citation analysis, an expert feedback. We will gather topic information by using the OpenAlexAPI to pull this information matched by the DOIs in our final dataset. OpenAlex uses a hierarchical system that organises topics into levels ranging from broad to more specific. We will focus on lower level, more narrow fields (i.e., level 1 and beyond) that represent increasingly specific subfields within disciplines (e..g, ‘pediatric oncology’, ‘telemedicine’, ‘anxiety’). *(10) *We will report the 20 most frequent terms per year and if possible visualise this information using the VOS-Viewer free software. *(11) *”*

 Additionally, the Altmetric platform supports PubMed search queries. The authors might want to explain why they prefer API for data retrieval instead of the user interface.

**Response: **To clarify, we did use the user interface. The initial phrasing relating to the API was to allow us to explore if this was a good approach for our purposes. Previous literature has used the API to pull based on article-level information such as DOI or keywords but after discussions with Altmetric, it was confirmed that pulling data based on an organisations GRID was not feasible with our licensing access. As the focus of our project was on the organisation level, PubMed search queries would not be appropriate in our circumstance. We have edited and added text to the dataset section to clarify this: “ *We will use Altmetric Explorer to search for all research outputs published between 1 January 2017 and 31 December 2023 from active Irish organisations that have Research Organisation Registry (ROR) IDs. ROR is a global registry of open persistent identifiers for research organisations which helps link researchers and their outputs to institutions across sectors (e.g., education, government, healthcare, non-profit, etc.) (https://ror.org/about/). Altmetric uses the prior system, the Global Research Identifier Database (GRID) (https://www.grid.ac/) which maps to ROR. As of 9 April 2024, there were 663 active research organisations with Ireland listed as their country of address. We searched both active and inactive IDs in case an organisation had outputs during the time period but then decided to inactivate their indexing GRID (e.g., they published in 2017-2019 but then deactivated their ID). The lead author (MKS) and two medical students will use the Altmetric Explorer interface to search for research outputs published within our date frame from each individual organisation. Datasets will be tracked using a tracking log in Excel to record the downloader (initials), total number of research outputs, number of outputs mentioned, filename, and download date. If an organisation has a least 1 output, we will download their data Altmetric uses the prior system, the Global Research Identifier Database (GRID) (https://www.grid.ac/) which maps to ROR.* *If an organisation has a least 1 output, we will download their data as a csv file using a standard naming notation (ID_YYYY-MM-DD). These csv files will be stored in one folder, spot checked for completeness (MKS), then combined into one dataset which will include research output data from all organisations producing output from 2017 - 2023.* *Of note, the datasets have the same 46 variables, will be downloaded in UTF-8 to account for non-English characters, and dates will be checked prior to stacking. This tracking log and our RMarkdown code detailing combining of datasets, cleaning, pre-processing, and more can be available on our Open Science Framework accompanying our results manuscript.(10) While the Altmetric API (https://www.altmetric.com/solutions/altmetric-api/) is also available for information retrieval, we did not find it suitable for pulling data based on an organisation’s GRID ID.”*

 Altmetric.com recommends using an Altmetric attention score of 20 as a benchmark. The authors did not mention this for quantitative analysis.

**Response:** We have added a comment within the analysis section to address this: *“* *We will also report the number of outputs with a score of 20 or above as Altmetric has indicated that this is a general score which can be considered as doing better than most of its ‘colleagues.’* *(12)* *”* As this score can vary by field and is not necessarily an indicator of quality, we do not want to relay or overemphasize its importance by integrating it into more detailed analyses. 

 4. Limitations: The manuscript could benefit from addressing potential biases in Altmetric data collection and how these might affect the study's findings.

**Response: **We have elaborated upon the limitations, potential biases, and our efforts to address them.

*“The lack of a links between preprints (manuscripts uploaded to databases without peer review) and postprints is a larger issue (peer reviewed journal articles) within academic publishing* *(13)* *as each item is assigned a unique DOI and there are challenges indexing pre-prints alongside their peer-reviewed publication. Of note, in the Altmetric dataset, pre-prints are included and tracked, they just have their own individual altmetrics separate from the final publication. Pre-prints played an unprecedented role in disseminating Covid-19 research, *(14) *so we will include them in our dataset, account for this in statistical analyses, and explicitly disclose this pre-print prevalence in our dataset, and frame our findings with this in mind.” …*

*“Altmetric data has also been noted to be prone to manipulation and artificial inflation (16) and some sources are particularly unstable, with certain items ‘vanishing’. (3) We will try to address this by pulling the data in a discrete period of time and we have included a time buffer (i.e., the end of 2023). However, we do recognise that certain mediums may still be ‘incomplete’ as they have different trajectories of attention growth – for example, Twitter attention starts and ends quickly, Mendeley readers accumulate quickly but continue to grow over the years, and policy attention is the slowest form of impact to accumulate. (17)”*

*“Furthermore, a high AAS does not necessarily indicate a high-quality piece of research.*(15) *Retracted publications have been widely shared online and certain topics (e.g., applied research, lifestyle behaviours, pop psychology, etc.) often simply receive more attention.*(16) *However, recent work comparing altmetrics to norm-referenced peer review scores from the UK Research Excellence Framework 2021 found that Altmetric correlated more strongly with research quality than previously thought although there is large variability with the strength of correlations amongst mediums and between fields (e.g., stronger in health and physical sciences than in the arts and humanities). *(3) *”*

 Specific Comments:

 The statement "With around 1.5 million new items being added to PubMed per year, or 2 papers per minute..." could include a reference to the source of this statistic for credibility.

**Response: **The reference for this statement is at the end of the sentence. We have moved it to clarify that it is the source of the information.

 The sentence "Ireland can also be consider an outlier in other ways..." has a grammatical error ("consider" should be "considered").

**Response: **We have fixed this typo.

 Overall, the manuscript presents a potentially valuable contribution to understanding online attention to health research in Ireland. However, the research protocol needs improvement, particularly in the areas of method design and justification.

**Response: **Thank you for this comment. We have attempted to address the methodological concerns in the response to the prompts above. Thank you for your helpful suggestions!

# Reviewer report: Reviewer response for version 1

**DOI:** 10.21956/hrbopenres.15238.r40897

**Published:** 2024-06-27

**Authors:** Dimity Stephen

**Affiliations:** 1 German Centre for Higher Education Research and Science Studies (DZHW), Berlin, Germany

The protocol presents the planned method for conducting a cross-sectional study that explores the online attention received by medical/health-related research published by institutes in Ireland in 2017-2023, particularly in the context of the COVID pandemic. The study seeks to identify how much attention is received and via which channels, disambiguated by sector, research institute, field, research topic, and study design type over time. The study will also assess the correlation between altmetric attention and citations. The methodology is sound and the sources of data are reputable and reliable. The authors also thoughtfully outline the limitations of their study and its potential findings. However, further details about specific aspects of the methodology could be given and attention should be given to citation windows (detailed below). 

 Specific comments:

 Are you planning to restrict the content to a particular document type, e.g., journal article, or any published item is within scope? 

 The data collection will produce a very large amount of information -- 7 years of daily counts of altmetric events for thousands of publications from over 600 institutions. You could provide more information about how exactly you will analyse this data to identify meaningful trends in attention between channels, fields, institutions, etc, and what statistical comparison will be made. A key point here is that altmetric data are rife with zero counts. Any statistical modelling will need to take this into account. 

 "Assuming an adequately sized dataset, we will also like to investigate whether Altmetric Attention Scores correlate to traditional article-level citation metrics, we will use Crossref’s metadata, the rcrossref package, and the Crossref API (https://api.crossref.org/) to match outputs by DOI to obtain data to run Pearson’s correlation tests on the data." Please keep in mind that you need to allow a citation window during which point citations accrue to a level that represents the articles' likely long-term impact. This is usually 3 years, so it would be best to analyse only articles published 2017-2020. Similarly, this window should be applied to each publishing year, e.g., for items published in 2017, citations that occur within 2017-2019 are included, for 2018, citations 2018-2020, and so on. Otherwise articles published in 2017 cannot be reliably compared with articles published in 2022, because 2017 articles have had sufficiently longer to accrue citations. Applying a stable citation windows means citation counts between publication years are comparable. This is less important for Altmetric Attention Scores, because most attention occurs shortly after publication. 

 "Study design. Given an appropriate size of the dataset, we will first use the metadata available in Crossref dataset to pull the research output’s abstract and author keywords which will then undergo classification. We will create a list of key terms, in consultation with our project’s steering committee and medical librarians and to classify research outputs within the umbrella categories of: intervention studies (e.g., trials), observational studies (cohort, cross-sectional, case control, genetic association, DTA, etc.), qualitative research (focus groups, interviews), protocols, case reports, evidence syntheses (e.g., systematic and scoping reviews), editorials or opinions, and other. These likely will be linked to the stopwords created during topic modelling. At least one reviewer will check the classifications after review."

 I'm a little confused about this section. It initially sounded as if you intended to manually classify the documents into categories. However, reading again, it sounds like the key terms will be developed by the steering committee and librarians and then perhaps some kind of automated classification process will be applied here. Could you please clarify how these documents will be classified? If this is to be done manually, this could consist of classifying thousands of documents and is quite a heavy workload. Also, in the last line "These likely will be linked to the stopwords created during topic modelling", do you perhaps means topics rather than stopwords? 

 Finally, care should be given in interpreting these data and the weight attributed to altmetric attention, particularly in the context of research assessment. Applied research and particularly "hot" topics that are trending or interesting to a general population, such as pop psychology and diet- and sleep-related topics, may draw more attention than topics from base research that are less accessible and applicable to a general public. However, that does not necessarily mean they are more valuable. Please consider this when interpreting the results and making recommendations. 

 A couple of formatting/language things:

 Abstract: "reach can cannot be solely"

 Introduction: "(e.g., Stack Overflow), and more. (https://www.altmetric.com/about-us/our-data/our-sources/)" -> fullstop after the closing bracket of the reference 

 Methods: "The ANZSRC is a hierarchical system ... statistics in Australia and New Zealand.(19)" Is 19 a reference? It doesn't appear to match the content for reference 19. 

 Analysis: "a daily quarterly, and yearly basis" -> daily,

Is the study design appropriate for the research question?

Yes

Is the rationale for, and objectives of, the study clearly described?

Yes

Are sufficient details of the methods provided to allow replication by others?

Partly

Are the datasets clearly presented in a useable and accessible format?

Not applicable

Reviewer Expertise:

Scientometrics, bibliometrics

I confirm that I have read this submission and believe that I have an appropriate level of expertise to confirm that it is of an acceptable scientific standard.

# Response

**Published:** 2024-08-19

**Authors:** Melissa Sharp

**Affiliations:** Royal College of Surgeons in Ireland, Ireland

The protocol presents the planned method for conducting a cross-sectional study that explores the online attention received by medical/health-related research published by institutes in Ireland in 2017-2023, particularly in the context of the COVID pandemic. The study seeks to identify how much attention is received and via which channels, disambiguated by sector, research institute, field, research topic, and study design type over time. The study will also assess the correlation between altmetric attention and citations. The methodology is sound and the sources of data are reputable and reliable. The authors also thoughtfully outline the limitations of their study and its potential findings. However, further details about specific aspects of the methodology could be given and attention should be given to citation windows (detailed below).

**Response:** Thank you for the time and energy spent reviewing our protocol and for asking thoughtful questions and giving constructive feedback. We have attempted to clarify and elaborate upon our methodological and statistical approaches through our response below. We have also taken your feedback regarding the citation windows on board and have addressed it in the in further detail below.

 Specific comments:

 Are you planning to restrict the content to a particular document type, e.g., journal article, or any published item is within scope?

**Response: **We have added additional information regarding included ‘research outputs’/document types within the dataset section. An overwhelming majority of research outputs will be journal articles but we will not exclude other outputs (i.e., book chapters or books). *“Research outputs included are largely journal articles although book chapters and books will also be included. Of note, some items within Altmetric are classified as ‘news’ but can be considered articles as they are perspectives, commentaries, overviews, hot topics.”*

 The data collection will produce a very large amount of information -- 7 years of daily counts of altmetric events for thousands of publications from over 600 institutions. You could provide more information about how exactly you will analyse this data to identify meaningful trends in attention between channels, fields, institutions, etc, and what statistical comparison will be made. A key point here is that altmetric data are rife with zero counts. Any statistical modelling will need to take this into account.

**Response:** The note about a potentially large prevalence of zero count scores is important to consider, particularly for the Altmetric Attention Score (AAS) and mediums. While a majority of our analyses are descriptive in nature (i.e., counts and percentages), a large number of 0 counts could influence things like measures of central tendency so we will run analysis on both the full (unique deduplicated) dataset as well as on a dataset which excludes the 0 counts to provide a more complete view of our dataset. We have added text regarding this in the analysis section: “ *For Altmetric analyses, we will report counts, and averages and medians per medium (e.g., X, Facebook, policy documents) for both the deduplicated dataset as a whole and for the deduplicated dataset with the zero-count AAS removed as there may be large amounts of zero counts in our dataset, potentially skewing measures of central tendency.”*

 For any statistical comparisons, we will use zero-inflated negative binomial regression to account for metrics with excessive zeros and likely a quite dispersed outcome variable (in this case citation counts). We have added information in the analysis section addressing this: *“* *We will use zero-inflated negative binomial regression to account for the large number of AAS scores of zero.”*

 "Assuming an adequately sized dataset, we will also like to investigate whether Altmetric Attention Scores correlate to traditional article-level citation metrics, we will use Crossref’s metadata, the rcrossref package, and the Crossref API (https://api.crossref.org/) to match outputs by DOI to obtain data to run Pearson’s correlation tests on the data." Please keep in mind that you need to allow a citation window during which point citations accrue to a level that represents the articles' likely long-term impact. This is usually 3 years, so it would be best to analyse only articles published 2017-2020. Similarly, this window should be applied to each publishing year, e.g., for items published in 2017, citations that occur within 2017-2019 are included, for 2018, citations 2018-2020, and so on. Otherwise articles published in 2017 cannot be reliably compared with articles published in 2022, because 2017 articles have had sufficiently longer to accrue citations. Applying a stable citation windows means citation counts between publication years are comparable. This is less important for Altmetric Attention Scores, because most attention occurs shortly after publication.

**Response: **Thank you for raising this important concern, we will only have whole citation count data (not split by citations per year), so to account for this we will split our dataset by year and only perform analyses for the 2017-2020 years. We have edited the text accordingly to describe these changes: *“As articles in 2017 have had more time to accrue citations than those published in 2023, we will restrict our analyses and split our dataset by year. Previous research has indicated that a 3-year citation window is relatively stable so we will perform these analyses on the data from 2017-2020 only. (17,18)”*

 "Study design. Given an appropriate size of the dataset, we will first use the metadata available in Crossref dataset to pull the research output’s abstract and author keywords which will then undergo classification. We will create a list of key terms, in consultation with our project’s steering committee and medical librarians and to classify research outputs within the umbrella categories of: intervention studies (e.g., trials), observational studies (cohort, cross-sectional, case control, genetic association, DTA, etc.), qualitative research (focus groups, interviews), protocols, case reports, evidence syntheses (e.g., systematic and scoping reviews), editorials or opinions, and other. These likely will be linked to the stopwords created during topic modelling. At least one reviewer will check the classifications after review."

 I'm a little confused about this section. It initially sounded as if you intended to manually classify the documents into categories. However, reading again, it sounds like the key terms will be developed by the steering committee and librarians and then perhaps some kind of automated classification process will be applied here. Could you please clarify how these documents will be classified? If this is to be done manually, this could consist of classifying thousands of documents and is quite a heavy workload. Also, in the last line "These likely will be linked to the stopwords created during topic modelling", do you perhaps means topics rather than stopwords?

**Response:** Thank you for this query. Due to the resource constraints noted, the size of the dataset, and the quality of keywords/tagging, we have removed the section relating to study design part of the secondary objective and the objective text relating to that approach.

 Finally, care should be given in interpreting these data and the weight attributed to altmetric attention, particularly in the context of research assessment. Applied research and particularly "hot" topics that are trending or interesting to a general population, such as pop psychology and diet- and sleep-related topics, may draw more attention than topics from base research that are less accessible and applicable to a general public. However, that does not necessarily mean they are more valuable. Please consider this when interpreting the results and making recommendations.

**Response:** This is an important distinction which we have aimed to elaborate on in further detail in the Limitations section: *“* *Furthermore, a high AAS does not necessarily indicate a high-quality piece of research.(23) Retracted publications have been widely shared online and certain topics (e.g., applied research, lifestyle behaviours, pop psychology, etc.) often simply receive more attention.(24) However, recent work comparing altmetrics to norm-referenced peer review scores from the UK Research Excellence Framework 2021 found that Altmetric correlated more strongly with research quality than previously thought although there is large variability with the strength of correlations amongst mediums and between fields (e.g., stronger in health and physical sciences than in the arts and humanities). (5)” *We will also keep this distinction in mind for our results manuscript.

 A couple of formatting/language things:

 Abstract: "reach can cannot be solely"

 Introduction: "(e.g., Stack Overflow), and more. (https://www.altmetric.com/about-us/our-data/our-sources/)" -> fullstop after the closing bracket of the reference

 Methods: "The ANZSRC is a hierarchical system ... statistics in Australia and New Zealand.(19)" Is 19 a reference? It doesn't appear to match the content for reference 19.

 Analysis: "a daily quarterly, and yearly basis" -> daily,

**Response: **Thank you for pointing out these errors. We have addressed and fixed all noted problems. We have removed ‘daily’ as due to the size of the dataset this granularity was deemed unnecessary as it did not add much additional information.
